# Does human cytomegalovirus provide a novel therapeutic target for patients with glioblastoma?

**DOI:** 10.1098/rstb.2024.0403

**Published:** 2025-11-06

**Authors:** Cecilia Söderberg-Naucler, Mattia R. Pantalone, Giuseppe Stragliotto, Jiri Bartek

**Affiliations:** ^1^ Department of Medicine, Solna, Karolinska Institutet, Stockholm 17176, Sweden; ^2^ Institutet of Biomedicine, University of Turku, Faculty of Medicine, Flagship InFLAMES, Turku 20520, Finland; ^3^ Department of Neurology, Karolinska University Hospital, Stockholm 17176, Sweden; ^4^ Department of Neurosurgery, Karolinska University Hospital, Stockholm 17176, Sweden; ^5^ Danish Cancer Institute, Danish Cancer Society, Copenhagen DK-2100, Denmark; ^6^ Department of Medical Biochemistry and Biophysics Solna, Division of Genome Biology, Science for Life Laboratory, Karolinska Institutet, Stockholm 17176, Sweden; ^7^ Czech Academy of Sciences, Institute of Molecular Genetics, CZ-142 20 Prague, Czech republic

**Keywords:** glioblastoma, cytomegalovirus, reactivation, treatment, valganciclovir, vaccine

## Abstract

Glioblastoma is the most common and aggressive primary malignant brain tumour in adults, with limited treatment options despite years of research. Since the 2005 introduction of the current standard of care, hundreds of clinical trials have failed to deliver significant breakthroughs. In 2002, human cytomegalovirus (HCMV) was detected in 100% of glioblastoma tumours. Although its role in cancer remains debated, and HCMV is not classified as an oncovirus, numerous studies have reported high viral prevalence in glioblastoma. HCMV can induce all 10 ‘hallmarks of cancer’ and has been shown to modify both tumour cell behaviour and the microenvironment, which may enhance tumour growth and promote immune evasion. The association between HCMV and poor glioblastoma prognosis has generated increasing interest in targeting the virus therapeutically. Our clinical studies suggest that adding antiviral treatment to standard care may improve survival in both primary and recurrent glioblastoma. Moreover, an mRNA-based HCMV pp65 dendritic cell vaccine has shown preliminary indications of a potential survival benefit in early phase studies. Future research should prioritize clarifying HCMV’s role in glioblastoma and rigorously evaluating antiviral and immunotherapeutic strategies in randomized clinical trials.

This article is part of the theme issue ‘The indirect effects of cytomegalovirus infection: mechanisms and consequences’.

## 1. Introduction

Glioblastoma is an incurable and highly aggressive brain tumour with a poor prognosis. With an incidence rate of 3.21 per 100 000 people annually (American Association of Neurological Surgeons, see https://www.aans.org/patients/conditions-treatments/glioblastoma-multiforme/), glioblastoma is the most common malignancy of the central nervous system in adults, accounting for approximately 50% of all malignant brain tumours. The median age at diagnosis is 60 years, and the disease is more prevalent in men than in women. Unfortunately, there is no curative treatment for glioblastoma. Standard therapy involves an initial surgical resection, as complete as possible, followed by fractionated radiotherapy (total dose of 60 Gy over 6 weeks) and concurrent chemotherapy with temozolomide (TMZ) [1,2]. Achieving complete tumour resection significantly improves survival outcomes [1,3], with patients undergoing more than 90% tumour removal showing higher 1-year survival than those with less extensive resections [4]. The smaller the residual tumour remaining after surgery, the better is the survival observed [5], highlighting the critical importance of optimizing surgical removal when feasible.

Despite advancements in understanding the molecular and genetic features of glioblastoma, the prognosis remains grim [6]. The tumour rapidly develops resistance to therapy, recurs and ultimately leads to patient death. The 2-year survival rate is approximately 15−26%, with a median overall survival (OS) of only 12−15 months [7], a figure that has not significantly improved in recent decades. Since 2005, over 400 clinical trials have explored novel therapies for glioblastoma [8], yet only a few have shown meaningful survival benefits [9,10]. US Food and Drug Administration (FDA)-approved treatments for glioblastoma include TMZ, bevacizumab and Optune [9,11,12]. Bevacizumab lacks approval in Europe, and Optune is not universally available despite European approval. However, despite all aggressive treatment strategies that have been evaluated in clinical trials, including immune checkpoint inhibitors that are effective in other types of cancer, glioblastoma patients still face a lack of effective treatment options.

## 2. The aetiology of glioblastoma is unknown

The primary reason for the failure to improve patient prognosis is likely the lack of understanding of the underlying causes of glioblastoma. These tumours are highly vascularized, invasive, rapidly proliferating, and display pronounced genetic instability [13]. The prognosis is influenced by multiple factors, including patient age, comorbidities, pre-operative and post-operative performance status (cognitive and neurological function), extent of surgical resection [14], and methylguanine methyltransferase (MGMT) promoter methylation status [15]. However, with the exception of MGMT promoter methylation status (see below), none of these factors substantially alters survival outcomes. Although several environmental factors have been investigated, no direct risk factor has been identified, which is why glioblastomas are considered to be sporadic in nature.

Since 2021, the World Health Organization (WHO) has classified glioblastomas as isocitrate dehydrogenase (IDH)-wild-type diffuse gliomas [16]. The previously termed IDH-mutant glioblastomas and secondary glioblastomas are now classified separately as IDH-mutant, high-grade astrocytomas [16]. The pathological diagnosis of glioblastoma is based on histological features, such as mitotic index, necrosis, cellular hyperplasia and microvascular proliferation, in addition to molecular markers.

By integrating single-cell analyses, genetic data and lineage tracing, researchers have identified a model of cellular states and genetic diversity in malignant glioblastoma cells. This model includes four primary cellular states, each resembling distinct neural cell types: neural progenitor-like, oligodendrocyte progenitor-like, astrocyte-like and mesenchymal-like [17,18]. These states are influenced by copy number amplifications of the CDK4, epidermal growth factor receptor (EGFR) and platelet-derived growth factor receptor alpha (PDGFRα) loci, as well as by mutations in NF1 [17]. Gliomas also exhibit variability in cellular architecture, which corresponds to these cellular states and is reflected in established glioma stem cell markers [19]. Transitions between states are regulated by the tumour microenvironment (TME) and appear to have clinical prognostic relevance [17,19].

The methylation status of the MGMT promoter is routinely assessed at most centres and is a key predictor of a patient’s response to TMZ therapy [15]. Patients with an unmethylated MGMT promoter typically respond poorly to TMZ, as the MGMT enzyme is active and prevents the therapeutic effects of the drug. Approximately 50–60% of patients with glioblastoma have tumours with an unmethylated MGMT promoter [20,21], and their estimated 2-year survival rate is less than 10%. In contrast, patients with a methylated MGMT promoter tend to respond better to TMZ and have an expected 2-year survival rate of around 30% [15]. The expected median OS time is about 12.4−14 months in patients with unmethylated MGMT promoter status, compared with 21−25.5 months in patients with methylated MGMT [15,21,22].

## 3. The tumour microenvironment in glioblastoma: immune barriers and therapeutic opportunities

The tumor microenvironment (TME) in glioblastoma plays a central role in driving disease progression, treatment resistance and immune evasion [23]. The TME suppresses cytotoxic immune responses, limiting the activity of both natural killer (NK) cells and cytotoxic T lymphocytes [23]. Although NK cells can eliminate tumour cells via death receptors and cytotoxic granules, their function is often impaired by the dynamic expression of activating and inhibitory ligands on cells, changes driven by genomic instability and metabolic stress [24]. Similarly, T cells are suppressed within the TME through altered antigen presentation, upregulation of immune checkpoint ligands, and cytokine-mediated inhibition [25]. Tumour-associated macrophages (TAMs) and myeloid-derived suppressor cells (MDSCs) are central to this immunosuppressive environment [26]. They secrete interleukin (IL)-10, transforming growth factor beta (TGF-β), adenosine and reactive oxygen species (ROS), which impair NK cell cytotoxicity and hinder T cell proliferation, cytokine production and survival. Hypoxia and metabolic by-products, such as lactic acid, further contribute to T and NK cell dysfunction by promoting exhaustion and diminishing anti-tumour responses [27]. Glioblastoma cells also express inhibitory ligands like Programmed death1-receptor ligand (PD-L1), T cell immunoglobulin and immunoreceptor tyrosine-based inhibitory motif (ITIM) domain (TGIT), T cell immunoglobulin and mucin-domain containing-3 protein (TIM-3), which suppress immune cell activity by engaging their respective receptors on NK and T cells [28–30]. Additionally, signal transducer and activator of transcription 3 (STAT3) signalling within the TME reprogrammes these cytotoxic cells towards regulatory phenotypes, increasing CD73 expression and the release of immunosuppressive cytokines, thereby enhancing tumour immune evasion [31].

Despite these challenges, NK and T cells retain significant therapeutic potential. NK cells produce IFN-γ and tumor necrosis factor alpha (TNF-α), which enhance antigen presentation, promote T cell priming, and recruit dendritic cells (DCs) via chemokines such as X-C Motif Chemokine Ligand 1 (XCL1) and chemokine (CC motif) ligand 5 (CCL5) [32,33]. Similarly, properly activated T cells can mount sustained and effective anti-tumour responses. However, since the TME remains a critical barrier to successful immunotherapy, it is essential to enhance the anti-tumour capabilities of both NK and T cells. Strategies include adoptive transfer of activated or genetically engineered immune cells, immune checkpoint blockade and cytokine-based therapies [34]. Targeting immunosuppressive components of the TME, such as TAMs and MDSCs, may further synergize with these approaches to generate more effective and durable immune responses in glioblastoma.

## 4. Human cytomegalovirus infection in glioblastoma

In 2002, Cobbs *et al*. were the first to report that 100% of glioblastoma tumours are positive for human cytomegalovirus (HCMV) [35]. This prevalence is higher than expected in the general population, which is estimated at 83% [36]. Subsequent studies by many research groups have confirmed the frequent presence of HCMV proteins and nucleic acids in glioblastomas, as well as in other tumour types, such as colon, breast, prostate, ovarian cancer, medulloblastoma, neuroblastoma and sarcoma [37–48]. Our own research has also shown that HCMV is present not only in primary tumours but also in lymph node and brain metastases of colon and breast cancer [49,50]. Viral presence is often linked to an increased inflammatory phenotype in different types of tumours [44,51–54], and the activity of HCMV in the tumour seems to predict patient outcome [49,55–58].

However, while many investigators have confirmed the presence of HCMV in tumours, several studies have also failed to detect HCMV in tumours [59–64]. This has led to an ongoing debate in the field regarding the presence and role of HCMV in tumours, with no established scientific consensus in the scientific community. Some researchers remain unconvinced that HCMV affects tumours or patient outcomes in glioblastoma. To address this discrepancy, our team recently conducted a systematic review of 645 articles investigating HCMV in glioblastoma. We identified 81 studies that collectively performed 247 analyses on 9444 clinical samples, consisting of 7024 tumour samples and 2420 blood samples, using a variety of detection techniques. Optimized immunohistochemical methods identified HCMV in 84.2% of 1653 tumour samples, while non-optimized protocols showed much lower prevalence [65].

In addition to direct detection, other direct and indirect biological evidence supports the presence of HCMV in glioblastomas. For example, in patients receiving DC therapy using tumour lysates, a significant expansion of HCMV pp65-specific T cells has been observed [66]. This finding strongly implies that HCMV antigens are indeed present in glioblastoma tissue and capable of eliciting an immune response. These HCMV-specific T cells may, in theory, contribute to the positive treatment effects observed in patients undergoing DC vaccination with tumour lysates [10], although further controlled studies are needed to confirm this relationship.

Furthermore, Herbein’s team in France successfully obtained 11 HCMV strains from cultures of glioblastoma specimens and four strains from breast cancer samples, thereby confirming the presence of the virus in these tumours [67–70]. Intriguingly, these tumour-derived strains exhibited altered growth characteristics compared with reference laboratory strains and were classified as ‘high-risk’ because they were capable of transforming normal human astrocytes and mammary epithelial cells *in vitro* [67,69]. These findings suggest that some HCMV variants may possess unique biological properties, but their interpretation remains limited as the viral genomes of these isolates (except for HCMV-DB) have not been fully sequenced, and the strains themselves have not been made available for independent confirmation by other laboratories. Without such validation, it remains uncertain whether these findings reflect strain-specific properties or experimental artefacts.

Historical studies provide some additional support for a potential oncogenic capacity of individual HCMV genes. For instance, cloned fragments of the laboratory strain AD169 were shown to transform NIH 3T3 cells *in vitro* [71], raising the possibility that certain viral genes could influence malignant transformation. However, there is currently no strong evidence that intact clinical isolates of HCMV are able to independently transform normal human cells, and as a result, HCMV is not classified as an oncogenic virus [72]. Instead, the prevailing view is that HCMV acts in an oncomodulatory rather than carcinogenic manner, modulating key cellular processes in ways that may support tumour progression rather than directly causing tumour initiation.

From this perspective, it is likely that HCMV-encoded proteins play central roles in these oncomodulatory effects. For example, Herbein’s group detected viral proteins and nucleic acids in transformed cells and has sequenced at least one tumour-derived virus (HCMV-DB). Similarly, the Rapp group reported oncogenic properties of an HCMV isolate in the 1970s [73], but the strain could not be sequenced at that time, leaving its identity and relevance unresolved. Taken together, these observations raise the possibility that specific, yet-uncharacterized HCMV variants could possess oncogenic potential, but definitive evidence is lacking in the absence of full genomic characterization and confirmatory analyses.

Overall, while certain findings hint that some HCMV strains might harbour oncogenic properties, the weight of current evidence suggests that HCMV primarily acts as an oncomodulatory virus, influencing tumour biology without directly initiating malignant transformation. This interpretation is supported by data from solid organ transplant recipients, which show no overall increase in cancer risk among HCMV-positive donor/recipient pairs compared with HCMV-negative pairs [74,75]. However, it remains unclear whether HCMV reactivation in transplant recipients with existing malignancies worsens clinical outcomes, highlighting an important area for future investigation.

## 5. Human cytomegalovirus and its role in the human population

HCMV is a common β-herpesvirus that establishes lifelong latency and persistence after a primary infection. Although most infections are asymptomatic or mild in healthy individuals, the virus can cause severe disease in immunocompromised patients, such as those with acquired immunodeficiency syndrome (AIDS), or in recipients of organ or stem cell transplants [76]. A systematic review and meta-analysis published in 2019 estimated the global HCMV seroprevalence to be 83% (95% uncertainty interval (UI): 78−88) in the general population, 86% (95% UI: 83−89) in women of childbearing age and 86% (95% UI: 82−89) among blood or organ donors [36]. HCMV can be transmitted through all bodily fluids and is spread via close personal contact, blood transfusions and organ or stem cell transplants. HCMV reactivation and infection occur in 60−80% of transplant patients if no prophylactic measures are taken [77]. In immunocompromised individuals, HCMV infection can lead to retinitis (with a high risk of blindness), pneumonitis, gastroenteritis, hepatitis and an HCMV syndrome characterized by prolonged fever and fatigue [78]. To manage the infection, immunocompromised patients typically receive prophylactic antiviral treatment or follow a pre-emptive therapy protocol to detect and treat evolving HCMV infections early [78]. However, issues such as antiviral toxicity, the emergence of resistant strains and limited access to antiviral drugs in developing countries continue to make HCMV a sustained clinical challenge, placing this virus as a top priority for vaccine development [79–81]. In addition, HCMV can cause congenital infections, affecting 0.2−4% of newborns [79,80]. Among these cases, 15% exhibit clinical symptoms at birth, with some experiencing brain damage and/or hearing loss caused by the virus [79,80].

HCMV utilizes different receptors for entry into host cells. For infection of fibroblasts and neuronal cells, the virus targets platelet-derived growth factor receptor alpha (PDGFRα) and uses its glycoprotein complexes gH/gL/gO for entry [82,83]. In endothelial and epithelial cells, HCMV enters cells through its pentamer complex, which includes gH/gL, (unique long (UL)128, UL130 and UL131. Although the pentamer receptor remained unknown for decades, recent studies suggest that neuropilin-2 [84] and CD147 [85] may mediate infection in these cell types. In monocytes, HCMV induces signalling downstream of the epidermal growth factor receptor (EGFR) and integrin-mediated Src-family kinases [86,87]. Notably, both PDGFRα and EGFR genes are frequently over-amplified in glioblastoma, which may facilitate the virus’s entry into glioblastoma cells.

## 6. Human cytomegalovirus infection and its potential role in glioblastoma pathogenesis

**Figure 1.**
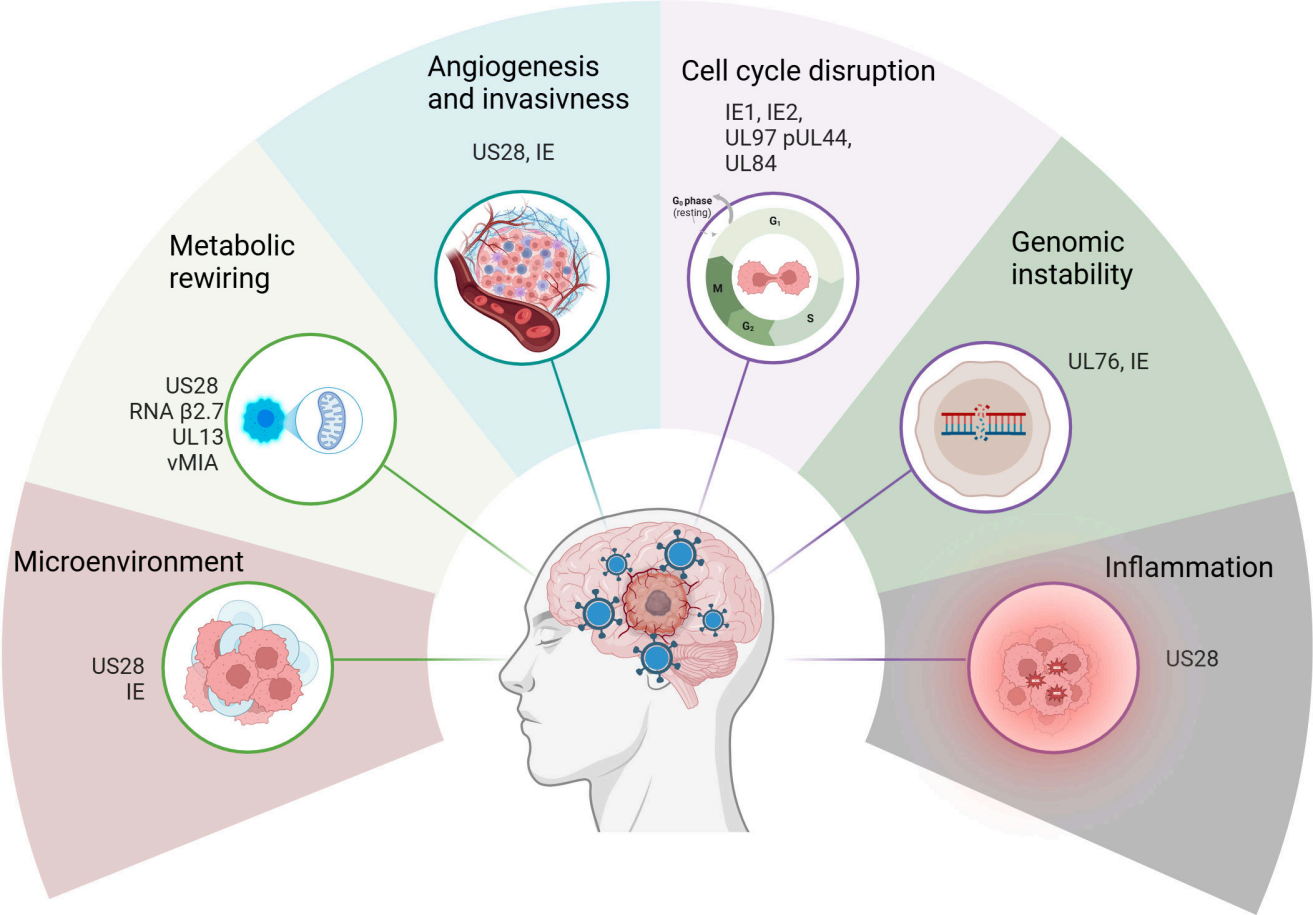
Human cytomegalovirus (HCMV) infection induces the 10 ‘hallmarks of cancer’. Different HCMV proteins and transcripts have been associated with the 10 hallmarks of cancer, and the most relevant and viral causative mechanisms implied are depicted in the figure. Created with Biorender.

The presence of HCMV in glioblastoma is intriguing not only because of its potential as a tumour marker for personalized therapy strategies but also because of its possible role in tumorigenesis. Several studies suggest that HCMV can contribute to glioblastoma progression by modulating key signalling pathways that promote tumour cell survival, proliferation, migration and immune evasion, and, in fact, HCMV can cause all 10 ‘hallmarks of cancer’ [88–90]. These hallmarks, first defined by Hanahan & Weinberg [91,92], represent the set of fundamental biological capabilities that enable tumour development and progression, including sustained proliferative signalling, resistance to cell death, angiogenesis, invasion, immune evasion and deregulated metabolism. The most relevant hallmarks related to HCMV’s oncomodulatory activity in glioblastoma are summarized in figure 1. HCMV is, however, not included among the group of defined oncogenic viruses [72]. To date, eight viruses have been classified as oncogenic, meaning they have the potential to cause cancer. These include human papillomavirus, Epstein–Barr virus, Kaposi sarcoma-associated herpesvirus, hepatitis B virus, hepatitis C virus, hepatitis D virus (HDV), human T-lymphotropic virus-1 and Merkel cell polyomavirus (MCPyV) [72,93]. Oncogenic viruses can transform cells through mechanisms that often involve targeting tumour suppressor proteins, such as the retinoblastoma protein (Rb) and p53, resulting in their inactivation [94–96]. These viruses also inhibit apoptosis and evade immune surveillance [93]. However, although HCMV can target and inactivate Rb and p53, inhibit apoptosis and evade immune surveillance, features commonly associated with oncogenic viruses, it does not appear to function as a classical oncogenic virus, but rather exerts a potential oncomodulatory role in cancer. The International Agency for Research on Cancer recently updated its evaluation of the carcinogenicity of HCMV, HDV and MCPyV and came to the same conclusion [72]. While both HDV and MCPyV were classified as ‘carcinogenic to humans’ based on ‘sufficient’ evidence, the Working Group was not unified in its assessment of HCMV. The group concluded that there is ‘limited’ evidence in humans that HCMV can cause acute lymphoblastic leukaemia, but the evidence was considered ‘inadequate for gliomas’. Mechanistic evidence supporting HCMV’s role in exhibiting key characteristics of carcinogens was judged ‘limited’ overall, although a small minority of the Working Group regarded it as ‘strong’. This divergence of opinion highlights the need for further studies to clarify HCMV’s role in cancer biology and, importantly, determine whether antiviral treatment could improve prognosis in affected patients.

## 7. Human cytomegalovirus oncomodulatory mechanisms

HCMV affects tumour suppressor proteins primarily through its virally encoded UL97 kinase, which phosphorylates and inactivates the Rb proteins p107 and p130; this process rescues E2F-dependent transcription [97–99], including genes involved in cell cycle progression, particularly those necessary for the G₁-to-S phase transition. In addition, the HCMV UL97 kinase promotes the degradation of Rb family proteins [100]. HCMV also uses its tegument protein, pp71, to bind to Rb family proteins and target them for proteasomal degradation [101]. This action further impairs cell cycle control and facilitates viral replication [102]. Furthermore, pp71 independently accelerates the G1 phase transition, bypassing its interaction with Rb proteins, thereby ensuring timely progression to the S phase, which is crucial for viral DNA replication [103]. As a result, HCMV-infected cells may enter a state conducive to uncontrolled proliferation, particularly in cancerous contexts, potentially exacerbating oncogenic processes. However, in normal cells, HCMV typically halts the cell cycle to create an environment conducive to viral gene expression, DNA replication and the production of new viral particles [104].

HCMV infection of normal cells forces them to halt in the G1/S-transition phase of the cell cycle, in order to utilize the cell’s machinery for DNA synthesis [104]. This process requires the manipulation of cellular functions to ensure that viral DNA replication takes precedence over that of the host, and the HCMV protein pUL117 is required to specifically block host DNA synthesis [105]. Other mechanisms through which HCMV achieves this include alterations in RNA transcription, modulation of cyclin-dependent kinases and other proteins involved in cell cycle regulation and DNA repair, and post-translational modifications that affect protein stability [106,107]. In addition, HCMV influences the ubiquitin-proteasome pathway, targeting specific proteins for degradation, and can even redirect cellular proteins to different locations within the cell to promote viral replication.

The HCMV immediate early (IE) add proteins IE1 and IE2, along with pUL44 and pUL84, regulate the tumour suppressor protein p53 [108,109]. These interactions lead to the stabilization of p53, but simultaneously repress its transcriptional activity, thereby preventing the activation of p53-responsive genes involved in cell cycle arrest and apoptosis. This repression may promote genomic instability and facilitate uncontrolled cell proliferation, but this effect may only occur in certain cell types (i.e. by affecting tumour cells differently from normal cells). For example, if IE proteins are expressed without pUL117, which normally inhibits cellular DNA synthesis, this may result in uncontrolled cellular proliferation. This phenomenon may occur only when tumour cells are infected by certain HCMV strains. HCMV can also activate various pro-oncogenic pathways, including those involving EGFR signalling, the phosphoinositide 3-kinase and the mitogen-activated protein kinase, as well as other kinase signalling pathways [110–113]. These pathways are frequently dysregulated in glioblastomas and are associated with increased tumour growth and resistance to therapy. HCMV infection also causes nuclear factor kappa-light-chain-enhancer of activated B cells (NF-κB) activation, leading to upregulation of the proto-oncogene c-mesenchymal-epithelial transition factor (*c-MET*) [114]. To enhance survival of infected cells, HCMV influences apoptosis regulation via several mechanisms [115–117], and in glioblastoma cells, in particular, inhibition of apoptosis was shown to be mediated by the activation of transcription factor 5, an anti-apoptotic protein that is upregulated in malignant gliomas and plays a key role in glioma cell survival [118].

HCMV replication is also dependent on the disruption of the host promyelocytic leukaemia (PML) bodies, a process that is mediated by the HCMV IE1 protein IE72 [119,120]. SOX2 facilitates HCMV gene expression in glioblastoma cells by downregulating PML and Sp100 expression, resulting in enhanced growth of neurospheres *in vitro* and xenografts implanted into immunodeficient mice. SOX2 and HCMV IE1 protein expression are correlated in clinical specimens, with higher expression associated with worse clinical outcomes [121]. Furthermore, HCMV infection in glioblastoma cell culture models enhances features of stemness, thereby increasing the glioblastoma stem-cell potential and promoting more aggressive tumour growth, which may in turn contribute to tumour progression [122,123]. Infection of glioblastoma cells or other cancer cells does not result in cell cycle arrest [124]; instead, we have observed increased proliferation on infection with the HCMV strain VR1814. We also showed that glucocorticoid treatment, which is often given to patients with glioblastoma, enhances the glioma stem-cell-like phenotype and confers resistance to chemotherapy in primary human glioblastoma cells [125]. Yet another, recently discovered tumour-promoting ability of either HCMV infection or even ectopic expression of the HCMV IE proteins is the capacity to trigger robust DNA replication stress and chromosomal instability in host cells [126], both of which are established as hallmarks of various types of cancer, including glioblastoma [126–131]

We found that HCMV infection, or ectopic expression of the HCMV IE gene in human tumour cells, slows replication fork progression in host cells, leading to robust replication stress accompanied by DNA damage, mitotic errors and chromosomal instability [126]. This instability is a hallmark of cancer and is associated with tumour aggressiveness, therapy resistance and poor prognosis. We also observed this cell-cycle–slowing effect in epithelial-derived cancers, such as colon and breast cancer (unpublished observations, 2025). HCMV-induced replication stress and chromosomal instability have also been reported in our studies in various human tumour cell lines, including glioblastoma [126]. In breast and colorectal cancer models, we found that HCMV infection promotes epithelial-to-mesenchymal transition (EMT) and metastasis (unpublished observations). In contrast, in glioblastoma cells, we have observed increased proliferation of infected cells.

It is possible HCMV might fuel cancer progression in pre-cancerous or nascent tumour cells (i.e. those mostly absent from the bulk of transplant patients) owing to the major, and so far mechanistically unexplained, difference in how normal human cells versus cancer cells respond to HCMV infection: (i) normal cells are permissive and typically undergo the lytic cycle when infected by HCMV, leading to cell death. This precludes any HCMV-induced pro-tumorigenic effects from fully manifesting and progressing into tumorigenic transformation of these fully permissive cells. (ii) In sharp contrast, tumour cells infected with HCMV survive the infection. These cells are commonly non-permissive (unable to produce HCMV particles) or ‘semi-permissive’ (surviving HCMV infection while producing small amounts of viral particles). They can still express at least some HCMV genes and proteins, which leads to multifaceted phenotypic consequences reminiscent of cancer hallmarks [88–92]—yet without resulting in cell death (figure 1).

## 8. Human cytomegalovirus promotes angiogenesis and invasiveness

Glioblastomas are highly vascularized tumours, and HCMV has been implicated in the upregulation of several pro-angiogenic factors, including vascular endothelial growth factor (VEGF) and IL-8 [132,133]. *In vitro*, HCMV infection promotes angiogenesis [134]; however, this effect appears to be restricted to uninfected cells in the cultures, indicating an indirect influence of HCMV on angiogenesis, through the upregulation of factors such as VEGF in the viral secretome [135]. This process is partially mediated by the viral chemokine receptor homologue US28, which also promotes angiogenesis, tumour formation through the expression of cyclooxygenase-2 (COX-2) [136], and cellular invasiveness [137] (figure 1). These factors not only promote the formation of new blood vessels, supplying the growing tumour with nutrients and oxygen, but may also increase the permeability of the blood–brain barrier (BBB), facilitating tumour cell invasion and metastasis.

HCMV utilizes several mechanisms to enhance migration. We have shown that the viral chemokine receptor US28 induces migration of smooth muscle cells [137]. The expression of US28 in glioblastoma cells increases their growth potential, leading to the formation of larger tumours [138], which is mechanistically mediated through enhanced IL-6/STAT3 signalling. Glioblastoma cell migration may also be driven by HCMV-IE2 through upregulation of hnRNP A2B1 [139], a protein overexpressed in glioblastoma tumours. This upregulation, in turn, promotes alternative splicing of the récepteur d'origine nantais (RON) receptor tyrosine kinase, which is linked to cancer cell migration [140]. In addition, expression of HCMV-US28 has been linked to the upregulation of sphingosine-1-phosphate (S1P), a bioactive lipid that activates oncogenic signalling pathways in glioblastoma [141]. Enhanced S1P signalling promotes glioblastoma cell proliferation and survival by activating kinases such as AKT and CHK1, as well as transcription factors such as cMYC and STAT3. Moreover, HCMV increases the levels of cancerous inhibitor of protein phosphatase 2A (CIP2A), which contributes to multiple feed-forward signalling loops, further boosting glioblastoma cell proliferation and survival [141].

HCMV can also induce an EMT programme in glioma cells through the activation of the RIP2/NF-κB signalling pathway [142]. This leads to increased migration and invasion of glioblastoma cells, accompanied by increased expression of the mesenchymal markers N-cadherin and vimentin, and decreased expression of the epithelial marker E-cadherin. However, one study showed that HCMV causes the reverse process in glioblastoma and breast cancer cell lines: mesenchymal-to-epithelial transition [143]. These findings suggest that the effects of HCMV may vary between different cell types.

Importantly, these studies were conducted using wild-type HCMV, and there is currently no conclusive evidence that such strains are associated with cancer *in vivo*. Therefore, while these findings are informative, their relevance to cancer pathogenesis should be interpreted with caution, as cancer-associated HCMV strains, whose identities have not yet been fully revealed, may differ significantly from those typically studied.

## 9. Human cytomegalovirus affects cellular metabolism

One of the hallmarks of cancer includes the rewiring of cellular metabolism, which commonly occurs in cancer cells and is referred to as the Warburg effect. The Warburg effect was described by Otto Warburg in 1923, who discovered that cancer cells exhibit altered metabolism compared with normal cells [144,145]. In HCMV-infected cells, it was first recognized that infection resulted in enhanced mitochondrial DNA synthesis [146]. Later studies with more sophisticated tools showed that HCMV increases mitochondrial biogenesis, lipid metabolism, oxygen consumption rate and extracellular acidification rate in infected cells [147–149]. This rewiring of metabolism results in an enhanced ability for cells to produce biomass and energy, both of which are required for efficient virus production and for cancer cells to grow. Interestingly, it appears that the metabolic changes are essential for cancer cell growth. Nuclear transfer experiments have shown that the genetic information provided by the nucleus of cancer cells is not sufficient to cause cancer cell growth when the nucleus is transferred to a cell with normal metabolism [150]. However, if cancer cell nuclei are transferred to cells exhibiting the Warburg effect, cancer cell growth can occur. HCMV infection may therefore play a role in cancer metabolism by increasing mitochondrial respiration and glycolytic activity, as this virus rewires cellular energy pathways [151]. In glioblastoma cells, these alterations are associated with higher levels of ROS and lactate production, which affect both tumour cell growth and the TME [152]. HCMV US28 activates the HIF-1α/PKM2 axis in glioblastoma cells, resulting in increased VEGF and lactate secretion, as well as enhanced expression of HIF-1 target genes, including glucose transporter type 1 and glyceraldehyde-3-phosphate dehydrogenase, which are both involved in glucose metabolism [153]. The virus-encoded long non-coding RNA β2.7, a highly abundant RNA transcript, interacts with complex I of the mitochondrial electron transport chain to sustain ATP production in infected glioblastoma cells *in vitro* [154]. This interaction also prevents apoptosis through GRIM19, which is triggered by intrinsic stresses related to reduced ATP levels [154]. As rewiring of cellular metabolism interconnects HCMV with cancer cell growth, this may offer new therapeutic strategies [150,155]. In support of this statement, mice placed on calorie-restricted and ketogenic diets exhibit smaller tumours [155], and when a calorie-restricted diet was combined with a glutamine uptake inhibitor, glioblastoma tumours did not grow in these animals [156]. These observations suggest that restricting specific energy substrates can reduce glioblastoma cell growth. This represents an interesting new avenue for the treatment of glioblastoma patients, and several clinical trials are underway to explore the effects of metabolic intervention in cancer patients.

## 10. Human cytomegalovirus affects the tumour microenvironment

HCMV infection can also influence the TME by altering the behaviour of immune cells. In HCMV-positive tumours, viral protein expression is predominantly found in tumour cells, as well as in endothelial cells, pericytes and infiltrating macrophages, while normal tissue surrounding the tumours or their metastases is generally not infected. Microglia and macrophages, which make up a substantial portion of the glioblastoma cellular milieu, may contribute to the immunosuppressive environment of the tumour [157]. HCMV may promote the accumulation of immunosuppressive cells, such as MDSCs, M2-type macrophages and regulatory T cells (Tregs), which help maintain an immunosuppressive TME [157–160]. This may also enhance the angiogenic process in glioblastoma, as it has been shown that M2-macrophages act as bridging cells by localizing at the ends of two proximal tubules and promoting their fusion into a single, linked vessel [161]. Furthermore, HCMV has been reported to inhibit the function of tumour-infiltrating T cells, which are typically responsible for mounting anti-tumour responses. T cells are relatively scarce in glioblastoma tumours and often localize to the periphery of the tumour. This may further explain why glioblastomas, which typically show a low degree of immune infiltration, can evade immune surveillance despite the presence of tumour-specific and viral antigens that should trigger a T-cell-mediated response. Adding to the viral-induced immunosuppressive effects, higher PD-L1 levels are associated with HCMV in tumour specimens, and HCMV infection *in vitro* induces PD-L1 expression through toll-like receptor 3 (TLR3) regulation in glioblastoma, and this may promote a more malignant phenotype [162]. While this could represent a potential therapeutic target through PD-L1 blockade in selected patients [162], clinical trials have been less encouraging [163]. In contrast, NK cells, particularly NKG2C-positive adaptive NK (aNK) cells (also known as memory NK cells), are more frequently found within the tumour mass [164], and these cells can efficiently kill tumour cells, including glioblastoma cells [165]. This is interesting because NKG2C-positive NK cells are preferentially found in HCMV-infected individuals [166] and are known to resist the immunosuppressive effects of Tregs [167] and MDSCs in the TME [168], while being highly competent in controlling HCMV infections in virus carriers [32,169], while conferring high toxicity to tumour cells. Therefore, these cells are particularly interesting as potential candidates for immunotherapy in patients with glioblastoma [165].

## 11. Human cytomegalovirus reactivation and its role in glioblastoma progression

Recent data from our group and others demonstrate that HCMV can be reactivated in patients with glioblastoma who undergo brain radiation therapy. CD34^+^ haematopoietic stem cells are considered the primary site of viral latency in healthy human hosts [170], and the virus persists when these cells become monocytes in peripheral blood, while the viral genome is not maintained in circulating T and B cells. Reactivation is typically mediated by inflammatory responses, leading to the differentiation of myeloid lineage cells into pro-inflammatory macrophages or DCs [171,172]. Additionally, stress and DNA damage can trigger the reactivation of latent HCMV [126,173]. This is particularly relevant for patients with glioblastoma, as the DNA damage response (DDR) induced by brain radiation therapy can activate the HCMV major IE promoter via stress-induced transcription factors [126]. In experimental studies, we recently demonstrated that the DDR induced by radiomimetic drugs can activate the HCMV major IE promoter [126]. The latter phenomenon reflects the fact that the HCMV major IE promoter contains binding sites for a set of host-cell transcription factors that are normally employed to activate promoters of DNA repair and other human stress-response genes. Therefore, this recently discovered mechanism [126] suggests that HCMV can hijack the host-cell stress response for its own (re)activation, and may help explain the high prevalence of HCMV reactivation in GBM patients undergoing brain radiation therapy [57,174]. We suggest that through evolutionary adaptation, HCMV has developed a reliance on the activation of these cellular stress response pathways for its reactivation. What remains unclear is which cell types are the source of the reactivating virus in patients who develop clinical symptoms of HCMV reactivation following brain radiation therapy. Does the virus originate from infiltrating macrophages, from vascular endothelial cells or from the tumour cells?

Patients with glioblastoma who are seropositive for HCMV seem to have shorter OS [175]. The reason for this finding is not known, but prior exposure to the virus may define a group of patients at risk for viral reactivation. However, in this context, it should be noted that a substantial proportion of patients with HCMV-positive glioblastoma may be HCMV-seronegative [176,177]. Goerig’s team demonstrated that 45–48% of brain tumour patients receiving brain radiation therapy experience HCMV reactivation [57,174], with the highest incidence observed in elderly patients. These patients often become HCMV IgM-positive after radiation therapy, and 87% develop symptoms compatible with HCMV encephalitis. Elderly glioblastoma patients often receive reduced doses of radiation owing to concerns about cognitive and neurological side effects. It is possible that some of the cognitive symptoms these patients suffer from could be attributed to HCMV reactivation rather than solely to radiation-induced brain damage. Importantly, patients who were diagnosed early and treated with antiviral therapy showed a favourable response, whereas those who were not treated with valganciclovir experienced a poor outcome owing to HCMV encephalitis and rapid tumour recurrence [174]. Similarly, Ursu *et al.* found that 23% of HCMV-seropositive patients with glioblastoma developed HCMV reactivation at some point during treatment [178]. Older age, steroid intake and a low absolute lymphocyte count before radiotherapy were correlated with HCMV reactivation, and these patients had shorter OS [178]. In our own observations from the VIGAS trial, we found that 58% of placebo patients experienced IgM seropositivity upon radio–chemotherapy, indicating HCMV reactivation, while none in the valganciclovir group developed HCMV IgM positivity (unpublished observations, 2025). The risk of HCMV reactivation was associated with high steroid intake. Early tumour recurrences were frequent in HCMV IgM-positive patients, but did not occur in any of the valganciclovir-treated patients. These findings suggest that brain radiation therapy induces clinical HCMV reactivation in about half of glioblastoma patients, often causing encephalitis, which may then accelerate tumour progression. Importantly, this reactivation appears to be fully preventable with valganciclovir therapy, which also completely prevented early tumour recurrences in the small randomized VIGAS1 trial. These observations imply that infection is related to tumour progression and that valganciclovir may prevent both clinical reactivation of HCMV and neurological sequelae following brain radiotherapy and prevent viral-mediated tumour progression. Potentially, valganciclovir therapy could therefore improve the prognosis for brain tumour patients by reducing the negative effects of HCMV on the tumour and preventing early tumour recurrences.

## 12. Therapeutic implications of human cytomegalovirus in glioblastoma

**Figure 2.**
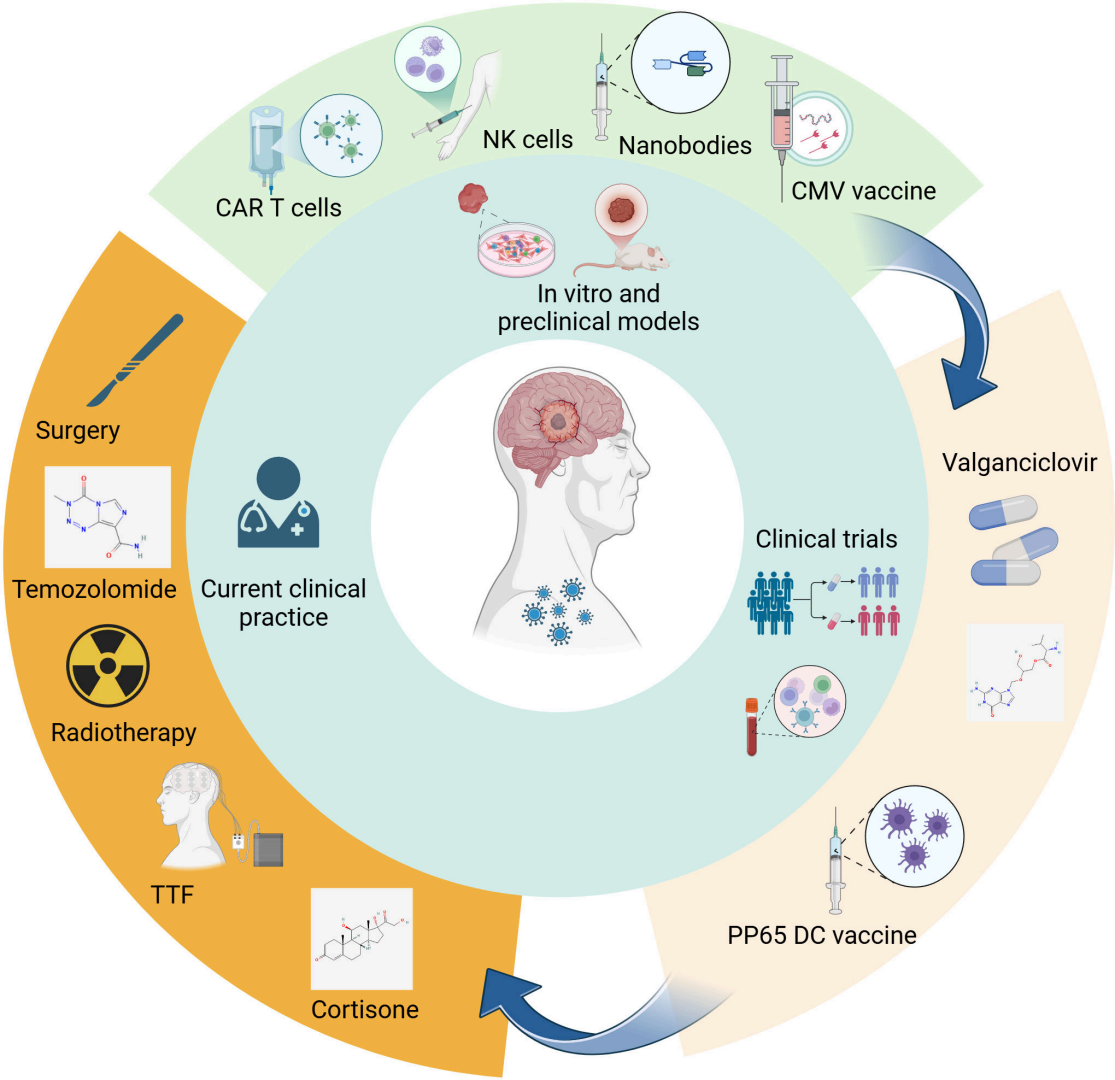
Current clinical practice in glioblastoma and evaluation of human cytomegalovirus (CMV)-directed therapies. Current standard of care includes surgery followed by Stupp regimen (and in many countries, also the recently introduced tumour-treating fields (TTF)). Cortisone is often used to relieve symptoms associated with oedema. Several therapeutic strategies, such as vaccines, US28-nanobodies, NK and CAR-T cell therapies, are currently being developed and are still in the pre-clinical phase, while clinical randomized trials are ongoing to evaluate valganciclovir and dendritric cell (DC) vaccines as add-on to the standard of care in phase II trials. Created with Biorender.

Given the widespread presence of HCMV in glioblastoma and its potential role in fuelling tumour growth, targeting this virus presents an exciting frontier in cancer treatment. Researchers are actively exploring several innovative approaches to harness HCMV as a therapeutic target, with the aim of integrating these treatments into future clinical practice (figure 2). One promising strategy revolves around repurposing antiviral drugs, such as valganciclovir, well known for treating HCMV infections in immunocompromised patients. This drug, which inhibits viral replication, could offer a unique adjunctive treatment for glioblastoma, particularly in patients who exhibit high viral loads, test positive for IgM or show evidence of HCMV infection within their tumour tissues.

## 13. *In vitro* and *in vivo* evidence supporting the use of anti-human cytomegalovirus therapy in glioblastoma

*In vitro*, we observed that primary tumour cultures from glioblastoma patients treated with the anti-HCMV drug ganciclovir showed a 90% reduction in HCMV protein expression, with a correspondingly high rate of tumour cell death. In animal models, anti-HCMV treatment reduced the growth of HCMV-positive medulloblastomas by 72% [41] and HCMV-positive neuroblastomas by 40% [40]. Valganciclovir had no effect on the growth of an HCMV-negative medulloblastoma [41], suggesting that its impact was virus-mediated rather than due to a general inhibition of cellular proliferation. Furthermore, in HCMV-infected and human GBM cells, ganciclovir impairs HCMV-driven growth phenotypes [122]. Additionally, treatment with the anti-HCMV drug cidofovir, or nanobodies targeting the HCMV protein US28, reduced tumour growth in human glioblastoma xenografts in mice [179,180]. These observations provide compelling evidence for the potential of anti-HCMV therapies in glioblastoma treatment.

## 14. Clinical evidence: valganciclovir improves prognosis in glioblastoma

We have examined over 400 glioblastoma cases using an optimized immunostaining technique. Only two cases were found to be HCMV-negative, and in most cases, HCMV protein expression was confined to tumour cells, with rare or absent detection in healthy tissue surrounding the tumours. Our analysis also revealed that patients with low-grade HCMV infection had a significantly longer OS compared with those with high-grade infection (33 versus 13 months, *p* = 0.036, HR: 2.2, 95% confidence interval (CI): 1.0−4.4). Two-year survival rates were 63.6% in the low-grade infection group versus 17.2% in the high-grade group (*p* = 0.003) [55]. Consistent with these early findings, a recent bioinformatic analysis of IGH-CDR3-CMV protein chemical complementarity suggested that stronger immune responses to HCMV, resulting in lower HCMV expression in tumours, may correlate with better survival outcomes [181].

All these findings highlight the possibility of using antiviral therapy against HCMV to reduce viral activity in glioblastoma and thereby improve patient outcomes.

We were the first to evaluate the effect of valganciclovir in a randomized, double-blind, phase I pilot trial (VIGAS, NCT00400322), initiated in 2006, to assess safety and estimate the potential efficacy of this therapy in patients with glioblastoma [182]. This pilot study was underpowered, aimed to aid in the design of a well powered future clinical trial, and the primary outcome of decreased tumour growth at 6 months was, as expected, not met among 42 patients, but we observed a trend towards reduced tumour growth (13.75 cm^3^ in controls versus 3.31 cm^3^ in valganciclovir-treated patients) [182].

While no significant difference in survival was observed in the intention-to-treat (ITT) analysis, the overall study cohort performed somewhat better than expected. In the ITT population, the median OS was 17.9 months (95% CI: 12.7−23.7) in the valganciclovir group and 17.4 months (95% CI: 11.7−22.5) in the placebo group (*p* = 0.4302, log-rank test), which is somewhat higher than typically reported for glioblastoma cohorts (12–15 months [1,7]). However, at six months, or earlier in the event of tumour recurrence, several patients had requested access to the active drug on compassionate grounds. Given ethical considerations, crossover was permitted, and ultimately 12 patients switched treatment (7 of them received valganciclovir for >6 months) and 7 patients in the valganciclovir group had also opted for valganciclovir treatment. Consequently, the ITT analysis for survival, a secondary endpoint, became difficult to interpret and essentially meaningless.

As the trial was meant to be hypothesis-generating, we performed an exploratory analysis that included all patients who received the drug, regardless of when treatment began, to further assess whether valganciclovir could have influenced survival. We used contemporary control patients from the same hospital, treated by the same physicians during the same time frame, to provide a robust comparison group with enough patients who had not received valganciclovir. It should be noted that all patients who opted for compassionate use (12 in the placebo group and 7 in the valganciclovir group) were doing well at the time of prescription, with stable or improved Karnofsky performance status (KPS) score compared with baseline, introducing potential selection bias. Moreover, this analysis is also inherently prone to immortal time bias and must be interpreted with caution. Nevertheless, patients who received at least six months of valganciclovir therapy had a median OS of 24.1 months, compared with 13.7 months in contemporary control patients (*p* = 0.0031, 95% CI: 6.9−17.3) [182].

The VIGAS1 trial remains the only completed randomized controlled study of valganciclovir in glioblastoma to date, and, as anticipated, it did not meet its primary endpoint. Nevertheless, it generated valuable survival data that formed the basis for power calculations for the ongoing VIGAS2 trial, which was initiated in 2019 after 9 years of efforts to secure funding.

During this period, additional patients received valganciclovir on a compassionate-use basis under our care, always initiated at the patient’s own request. A retrospective analysis of 50 patients treated with valganciclovir as an add-on to standard therapy demonstrated an unexpectedly high survival rate [183]. These analyses also included contemporary control patients for comparison. When interpreting survival data in glioblastoma, it is essential to consider the disease’s historically poor prognosis. Since 2005, more than 400 clinical trials have been conducted [8], yet OS curves remain remarkably similar and largely overlapping, regardless of treatment strategy. Meaningful patient selection is rarely possible in this setting, and no therapeutic intervention has so far demonstrated a substantial improvement in survival. For this reason, the inclusion of contemporary controls is particularly valuable in glioblastoma research, as they provide a more appropriate and realistic benchmark for comparison—a scenario that differs from many other cancer types where established standards of care lead to more predictable outcomes.

At 2 years, 62% of patients treated with valganciclovir were alive compared with 18% of controls (*p* = 0.001) [183]. The median OS in this cohort was 25.0 months, compared with 13.5 months in controls (*p* = 0.001, 95% CI: 18.7−37.4) [183]. In retrospective studies such as this, where the definition of exposure requires a minimum treatment duration, immortal time bias is a recognized concern. This bias arises when treatment initiation occurs in patients who are already doing well—for example, those who are tumour-free at the start of treatment—potentially leading to non-random selection and inflated survival estimates in the treatment group. By definition, patients must survive a certain period (the ‘*immortal time*’) without experiencing the outcome in order to be classified as exposed, which can artificially enhance apparent treatment effects [184].

To investigate whether this bias could explain the strong positive effect observed with valganciclovir, we performed a Cox regression analysis using treatment status as a time-dependent covariate. This analysis did not support the hypothesis that immortal time bias accounted for the observed survival benefit [185]. However, this re-analysis addresses only the bias resulting from misallocation of person-time and does not resolve other potential issues that arise when the start of follow-up and treatment allocation are not fully aligned. Furthermore, an intrinsic limitation of HRs is that they can be misleading when time-varying effects or selection bias are present, particularly when interpreting average or period-specific survival outcomes [186] . Since these data were analysed without modelling the intervention as a time-varying exposure, there is a potential risk of analytical bias, and the findings should therefore be interpreted with caution. In this context, we did not perform additional sensitivity analyses, such as landmark analyses, redefining the causal question, cloning followed by censoring or applying the plug-in g-formula [186,187].

In a subsequent 2020 study, the valganciclovir-treated cohort was expanded to 102 patients. The 2-year survival rate was 49.8% compared with 17.3% in 231 contemporary controls (*p* < 0.0001), and the median OS for the valganciclovir group was 24.1 months (identical to our first study) versus 13.3 months for controls [188]. Among patients receiving optimal therapy—defined in the VIGAS2 protocol as complete resection combined with concomitant radiotherapy and TMZ—OS was also longer in valganciclovir-treated patients (29.7 versus 17.0 months, *p* < 0.0001), and the 2-year survival rate was higher (63.9% versus 27.6%, *p* < 0.0001) compared with controls [188].

To assess whether immortal time bias could explain the increased survival observed in valganciclovir-treated patients, we performed a Cox regression analysis modelling treatment status as a time-dependent covariate. The resulting HRs from the two models—comparing time from surgery to treatment initiation versus untreated patients, and the original dataset—were not significantly different (HR = 2.238, 95% CI: 1.736−2.884 versus HR = 2.334, 95% CI: 1.813−3.003), reducing concerns that immortal time bias could explain the survival benefit [188]. However, the same methodological limitations discussed earlier still apply, and no additional analyses specifically addressing immortal time bias, such as landmark analyses, cloning with censoring or alternative causal modelling techniques, were performed in this study. Importantly, OS was longer in both MGMT-methylated patients (32.1 versus 15.15 months, *p* = 0.0006) and MGMT-unmethylated patients (21.1 versus 11.6 months, *p* < 0.0001) compared with controls [188]. This finding is particularly noteworthy, as patients with unmethylated MGMT promoter status typically have a poor prognosis and have never demonstrated such survival improvements.

Taken together, these results reinforce the potential significant survival benefit of valganciclovir as an adjunct therapy in patients with glioblastoma. While the Cox regression analysis reduces concerns that immortal time bias accounts for the findings, other sources of bias, including selection bias (e.g. patients requesting compassionate use may have had better performance status) and time-varying confounding, cannot be excluded. Therefore, the results should be interpreted with caution.

It is also noteworthy that very few patients treated with valganciclovir died within the first year after their diagnosis. Although this observation has faced criticism, we now believe that valganciclovir treatment may help prevent radiation-induced viral reactivation and early tumour recurrence. As reactivation appears to occur in approximately 50% of these patients; this mechanism could explain the consistent clinical outcomes observed across multiple analyses, showing that very few patients receiving valganciclovir die within the first year after diagnosis if they are treated with valganciclovir (with a mean time to treatment initiation from diagnosis of 2.7 months in the 102 patients examined).

## 15. Effect of valganciclovir on recurrent and secondary glioblastoma

We have also treated 29 patients with recurrent glioblastoma with valganciclovir as an add-on to second-line therapy. Recurrent glioblastoma is notoriously difficult to treat, with expected 2-year survival rates of only about 5%. Our data showed that valganciclovir treatment significantly improved the prognosis for recurrent glioblastoma patients. The 1-year survival rate was 44.8% in the valganciclovir-treated group, compared with 23.9% in controls (*p* = 0.0122), and the 2-year survival rate was 20.7 versus 5.5% in controls (*p* = 0.0042) [189] The median OS in the valganciclovir-treated group was 12.1 months, compared with 7.4 months in controls (*p* = 0.0028) [189]. We also treated eight patients with secondary glioblastoma, who previously had a low-grade glioma tumour that had progressed to glioblastoma. Likewise, patients treated with valganciclovir showed an increased median OS after progression to glioblastoma compared with controls (19.1 versus 12.7 months, *p* = 0.0072) [190].

Taken together, it is important to note that all these retrospective data are uncertain and may be subject to immortal time bias and unintentional selection bias; however, they consistently indicate that valganciclovir could potentially extend the life expectancy of patients with primary, recurrent and secondary glioblastoma. This is particularly noteworthy given that no other available treatment for glioblastoma has demonstrated a comparable improvement in survival, especially with a drug showing a very favourable tolerability profile. Therefore, it is important to conduct a randomized controlled study to evaluate the potential significant benefit of valganciclovir in patients with glioblastoma. We are currently conducting the VIGAS2 trial involving 220 patients to evaluate whether valganciclovir can prolong median OS time (ClinicalTrials.gov, identifier: NCT04116411). This trial was designed to evaluate the effect of valganciclovir therapy in patients with primary glioblastoma, with the sample size based on the retrospective cohort used for the power calculations. Given that MGMT promoter methylation occurs in approximately 30% of patients, enrolment of methylated cases was capped accordingly. Patient recruitment has now been completed, and study results are expected by the end of 2027. With current evidence indicating that a high proportion of glioblastoma patients undergoing radiotherapy experience HCMV reactivation and early recurrence, a process that appears to be largely preventable with antiviral therapy, the rate of HCMV reactivation may significantly influence outcomes. We propose that future clinical trials should account for this when developing study protocols.

## 16. Immune-based approaches to target human cytomegalovirus infection in glioblastoma

Beyond antiviral drugs, the use of immune-based therapies is also gaining traction for targeting HCMV in patients with glioblastoma. For instance, boosting the activity of HCMV-specific T cells *ex vivo* has shown some promise [191,192]. These T cells, armed to eliminate HCMV-infected tumour cells, have demonstrated cytotoxic effects, reducing glioblastoma growth in clinical settings in a few selected patients. However, NK cell therapies, particularly those involving NKG2C-positive adaptive or memory-like aNK cells, may offer a superior option [165], especially for HCMV-positive glioblastoma. aNK cells exhibit several distinctive features: they undergo clonal expansion in response to antigen exposure (particularly to HCMV-derived specific peptides), persist after pathogen clearance, mount recall responses to previously encountered antigens, resist suppression by MDSCs and Tregs within the TME and display strong cytotoxicity against tumour cells [193]. Unlike conventional NK cells, which are largely inhibited by NKG2A–HLA-E interaction, aNK cells express the activating receptor NKG2C. This receptor binds HLA-E in the presence of HCMV-derived peptides, such as UL40 or pp65 [169,194,195], and enables aNK cells to respond with a recall memory response. This places aNK cells at the intersection of innate and adaptive immunity, highlighting their relevance in infectious disease control, as well as in cancer immunotherapy. Glioblastoma tumour cells often upregulate HLA-E [196], and most glioblastoma tumours express HCMV peptides, which can trigger their recall memory response. Thus, their resilience in immunosuppressive conditions and sustained cytotoxic activity despite inhibitory signals make them strong candidates for developing more effective, durable and targeted therapies. By bypassing a key immune evasion mechanism, aNK cells therefore represent a promising frontier in glioblastoma immunotherapy.

Another exciting potential development is the use of DC vaccines utilizing pp65 mRNA [197,198]. Early trials suggest that this approach may enhance survival rates for glioblastoma patients, offering hope for a more effective treatment option [197,198]. In a phase I study, 11 patients with newly diagnosed glioblastoma were treated with a CMV pp65-pulsed DC vaccine in combination with intensified TMZ [197]. The trial reported a median OS of 41.1 months and a median progression-free survival of 25.3 months, compared with a cohort of matched contemporary controls, in whom the corresponding values were 19.2 and 8.0 months, respectively [197]. While limited by its small sample size and exploratory nature and the use of matched historical controls, the study provided data to inform the design of future trials. Several ongoing clinical trials are now evaluating pp65-loaded DC vaccination in patients with glioblastoma (NCT03927222 and NCT02465268). The latter of these studies has been completed and enrolled 175 patients; the results uploaded on ClinicalTrials.gov do not indicate a positive effect of the vaccine. Looking ahead, the development of targeted vaccines against HCMV may offer an approach to improving viral control in patients with glioblastoma in addition to antiviral therapy.

## 17. Challenges and future directions

While targeting HCMV in glioblastoma presents exciting possibilities, several challenges must be addressed. First, the specific strains of HCMV present in glioblastoma samples have not yet been identified with clarity, which may necessitate more tailored antiviral strategies. Additionally, because HCMV can establish latency and persist in host cells, effective treatment would need to target both actively replicating virus and latent reservoirs—an inherently difficult task. One promising strategy is to target the US28 molecule with antibodies or nanobodies, as US28 is expressed during both latent and active phases of HCMV infection, as well as in glioblastoma cells. Moreover, HCMV’s ability to evade and modulate the host immune system adds complexity to the development of effective therapies and should be considered in any therapeutic design. Another significant obstacle is the blood-brain barrier (BBB), which limits the delivery and efficacy of many systemic treatments. While some antiviral agents can cross the BBB, their therapeutic impact may be compromised by subtherapeutic concentrations within the tumour. Approaches such as nanoparticle-based delivery systems or convection-enhanced delivery may help overcome this barrier and improve treatment outcomes. Despite these challenges, ongoing research into the molecular and immunological aspects of HCMV in glioblastoma continues to offer hope for new therapeutic options. Finally, HCMV’s role in cancer metabolism remains a relatively unexplored but potentially fruitful area of study, with significant implications for identifying cancer-driving mechanisms and therapeutic targets. Advancing this knowledge could lead to treatments not only for glioblastomas but also for other tumours associated with HCMV, such as breast and colon cancer, where HCMV protein expression appears to be correlated with poor prognosis and shorter OS as well [49,56].

## 18. Conclusion

Glioblastoma remains one of the most challenging cancers to treat, leaving patients with a grim prognosis despite aggressive multimodal therapy. While significant strides have been made in understanding the molecular and genetic landscape of this disease, effective new treatments remain elusive.

Emerging evidence highlights the potential role of HCMV in glioblastoma progression. HCMV infection is found to be highly prevalent in glioblastoma when using optimised methods for virus detection. HCMV may contribute to tumour progression through various mechanisms, including immune evasion, angiogenesis, modulation of key signalling pathways, cell cycle control and alterations in cellular metabolism, suggesting a possible link between the virus and tumour biology.

Our research indicates that HCMV reactivation during brain radiation therapy may accelerate tumour progression, a process potentially preventable with antiviral therapy. Clinical data from our retrospective valganciclovir trials further support its potential to improve survival outcomes in patients with primary, recurrent and secondary glioblastoma, regardless of their MGMT promoter methylation status. Although these clinical data are encouraging, they should be interpreted with caution due to potential sources of bias, including the retrospective design of several analyses. Nevertheless, these findings, in our opinion, strengthen the hypothesis that HCMV plays a role in glioblastoma progression and underscore the need to explore antiviral therapies and immune-based strategies as innovative treatment options for patients with this devastating disease having no effective therapies. We propose that a deeper understanding of how HCMV influences glioblastoma pathogenesis and modulates the immune microenvironment will be essential for the development of targeted therapies aimed at improving patient outcomes.

However, many questions await answers: why is it so difficult to detect HCMV nucleic acids by PCR, and why can we not sequence this virus from tumour specimens, when *in situ* hybridisation methods and protein staining imply that the virus is there? Furthermore, why do optimised antigen-retrieval staining protocols need to be used to detect the virus in tumour specimens, when they are not required for HCMV detection in tissue specimens from patients with acute disease?

Taken together, current data suggest the possibility that HCMV, through its oncomodulatory properties, may contribute to glioblastoma progression. Consequently, targeting HCMV in glioblastoma patients remains an area of active investigation, although its clinical relevance has not yet been firmly established. In the future, combining antiviral strategies with immune-based treatments may offer a promising approach to improving therapeutic outcomes. However, robust evidence from well-designed randomised controlled trials will be essential before any definitive conclusions can be drawn regarding the role of HCMV-targeted therapies in glioblastoma management.

## Data Availability

This article has no additional data.
